# A Participatory Framework for Plain Language Clinical Management Guideline Development

**DOI:** 10.3390/ijerph192013506

**Published:** 2022-10-19

**Authors:** Rita Francisco, Susana Alves, Catarina Gomes, Pedro Granjo, Carlota Pascoal, Sandra Brasil, Alice Neves, Inês Santos, Andrea Miller, Donna Krasnewich, Eva Morava, Christina Lam, Jaak Jaeken, Paula A. Videira, Vanessa dos Reis Ferreira

**Affiliations:** 1 Associate Laboratory i4HB—Institute for Health and Bioeconomy, School of Science and Technology, NOVA University Lisbon, 2819-516 Caparica, Portugal; 2 UCIBIO—Applied Molecular Biosciences Unit, Department of Life Sciences, School of Science and Technology, NOVA University Lisbon, 2819-516 Caparica, Portugal; 3 CDG & Allies—Professionals and Patient Associations International Network (CDG & Allies-PPAIN), Department of Life Sciences, School of Science and Technology, NOVA University Lisbon, 2819-516 Caparica, Portugal; 4 Sci and Volunteer Program from NOVA School of Science and Technology/FCT NOVA, NOVA University Lisbon, Caparica, 2825-149 Setúbal, Portugal; 5 CDG Care, Colorado Springs, CO 80937, USA; 6 National Human Genome Research Institute, National Institutes of Health, Bethesda, MD 20814, USA; 7 Department of Clinical Genomics, Mayo Clinic, Rochester, MN 55905, USA; 8 Metabolic Centre, University Hospitals Leuven, 3000 Leuven, Belgium; 9 Department of Medical Genetics, Medical School, University of Pécs, 7622 Pecs, Hungary; 10 Division of Genetic Medicine, Department of Pediatrics, University of Washington School of Medicine, Seattle, WA 98195, USA; 11 Center for Integrative Brain Research, Seattle Children’s Research Institute, Seattle, WA 98101, USA; 12 Centre of Metabolic Diseases, KU Leuven, 3000 Leuven, Belgium

**Keywords:** clinical management guidelines, plain language, people-centric, congenital disorders of glycosylation (CDG), PMM2-CDG, rare diseases, participatory medicine, health literacy

## Abstract

Background: Clinical management guidelines (CMGs) are decision support tools for patient care used by professionals, patients, and family caregivers. Since clinical experts develop numerous CMGs, their technical language hinders comprehension and access by nonmedical stakeholders. Additionally, the views of affected individuals and their families are often not incorporated into treatment guidelines. We developed an adequate methodology for addressing the needs and preferences of family and professional stakeholders regarding CMGs, a recently developed protocol for managing congenital disorders of glycosylation (CDG), a family of rare metabolic diseases. We used the CDG community and phosphomannomutase 2 (PMM2)-CDG CMGs as a pilot to test and implement our methodology. Results: We listened to 89 PMM2-CDG families and 35 professional stakeholders and quantified their CMG-related needs and preferences through an electronic questionnaire. Most families and professionals rated CMGs as relevant (86.5% and 94.3%, respectively), and valuable (84.3% and 94.3%, respectively) in CDG management. The most identified challenges were the lack of CMG awareness (50.6% of families) and the lack of plain language CMG (39.3% of professionals). Concordantly, among families, the most suggested solution was involving them in CMG development (55.1%), while professionals proposed adapting CMGs to include plain language (71.4%). Based on these results, a participatory framework built upon health literacy principles was created to improve CMG comprehension and accessibility. The outputs are six complementary CMG-related resources differentially adapted to the CDG community’s needs and preferences, with a plain language PMM2-CDG CMG as the primary outcome. Additionally, the participants established a distribution plan to ensure wider access to all resources. Conclusions: This empowering, people-centric methodology accelerates CMG development and accessibility to all stakeholders, ultimately improving the quality of life of individuals living with a specific condition and raising the possibility of application to other clinical guidelines.

## 1. Introduction

Clinical management guidelines (CMGs) are “systematically developed statements to assist practitioner and patient decisions about appropriate health care for specific circumstances” [1]. CMGs make evidence-based recommendations on patients’ diagnosis, treatment, and follow-up. They also reduce the gap between research and clinical practice by establishing standard and flexible care protocols to guide health care professionals (HCPs) decision-making and promote clinical care consistency and quality [2,3].

Although methodological recommendations for professional CMG development and quality appraisal have been issued, the involvement of caregivers and patients has not been systematically established [2,4,5,6,7,8]. Additionally, professional CMGs, created mainly by and for clinical experts, are complex and use medical jargon, which hinders their comprehension and use by nonmedical stakeholders [1]. As a result, most family caregivers and patients struggle to understand them, particularly those with lower health literacy levels [9].

Health literacy is a constantly evolving construct defined as “people’s knowledge, motivation and competencies to access, understand, appraise, and apply health information in order to make judgments and take decisions in everyday life concerning healthcare, disease prevention and health promotion to maintain or improve quality of life during the life course” [10]. Health literacy interventions using plain language have shown positive results in health communication, comprehension, and usability by nonclinical stakeholders [9,11]. Plain language entails using simple, assertive, and clear words that nonmedical or scientific experts can understand [11].

The contribution of CMGs to accelerating diagnosis and improving patient clinical management is well recognised [3]. Consequently, international groups and institutions have listed CMG development as a priority, including the European Reference Networks (ERNs) which are rare disease networks dedicated to care and research [4].

The development of CMGs for rare diseases faces added difficulties due to the scarcity of high-quality data about patient clinical features and caregivers, clinical practices and patients’ needs and preferences [4,7]. These limitations contribute to the scarce availability of rare disease CMGs [2].

Congenital disorders of glycosylation (CDG) are an expanding family of rare metabolic diseases caused by genetic biosynthetic defects in glycosylation. They are primarily complex, multisystem diseases with predominant neurological involvement [12,13]. The most common type is phosphomannomutase 2 (PMM2)-CDG, primarily a neurological syndrome with additional age-specific manifestations encompassing liver, blood, immune system and/or heart dysfunction [14,15,16,17].

The CDG community needs have been identified, revealing a generalised proactive attitude among families towards health care decision-making and research participation. Moreover, the CDG community has expressed a high trust and reliance on patients’ organisations (POs) and digital solutions [2,3,18,19].

### Study Rationale and Aims

CMGs have been developed for some CDGs [20,21,22] based on professional experts’ revision and validation of the literature. Nevertheless, no family caregivers or patients were involved, so their feedback was not assessed, despite their status as potential CMG end-users. This gives cause for concern, and having these guidelines adapted to their preferences and needs is an important unmet need [3,18,19].

Hence, this work aims to promote the creation of a multistakeholder (i.e., encompassing CDG professionals and families), participatory and evidence-based framework to develop plain language CMGs, according to the principles of health literacy and the demands and preferences of the CDG community. Ultimately, this study aims to improve and accelerate the development of other plain language CMGs based on the CDG community and PMM2-CDG experience.

## 2. Methods

### 2.1. Development, Refinement, and Distribution of the CDG Journey Mapping Electronic Questionnaire

The CDG Journey Mapping Electronic (e-) Questionnaire is an author-built e-survey with two target audience-adapted versions. The first one was designed for people living with CDG and family caregivers. The second was designed for professionals (mainly HCPs, researchers, and limited numbers of pharmaceutical representatives).

**Figure 1:**
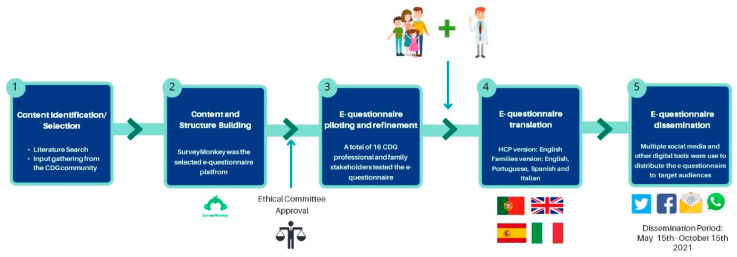
Development, refinement, and distribution of the CDG Journey Mapping e-Questionnaire.

The questionnaire content was designed to understand the entire CDG patient journey, ranging from diagnosis to information needs and health care/social support measures. The questions were based on (1) a literature review and identification of validated questionnaire-based tools and (2) input from members of the CDG community, including the Portuguese Association for CDG (APCDG) (Figure 1) [18,19,23,24,25].

Additionally, the e-questionnaire was piloted and reviewed by 16 families and professionals for comprehensibility and suitability to the target populations. Following e-questionnaire refinement, 96 and 79 questions were included in the family and professional versions, respectively. A section was specifically dedicated to assessing the CDG community’s views on CMGs (Supplementary Figure S1). This section included seven questions in the family version and eight in the professional version, with most questions being equivalent. This study included results from twelve CMG-related questions (five from the family and seven from the professional versions). Supplementary Figure S1 gives an overview of the CDG Journey Mapping e-Questionnaire, framing the CMG section. The e-questionnaire version for the professionals was only made available in English. In contrast, the version for the CDG patients and families was also translated to Spanish, Portuguese, and Italian to maximise participation (Figure 1).

Ethical approval for this study was granted by the ethics committee of the Faculty of Psychology, University of Lisbon, and e-informed consent was obtained from all participants. The e-questionnaire was created and administered through the SurveyMonkey Audience platform (www.surveymonkey.com/mp/audience (accessed on 31 March 2021)—Copyright#1999–2022 Momentive) (Figure 1). Respondents’ anonymity was ensured by blocking internet protocol (IP) identifier recording. The multiple-entry restriction feature was activated to prevent participant duplication. Different question formats, such as multiple-choice, matrix, and open-ended questions, were used. SurveyMonkey’s logic feature was added to specific questions to guide participants better and reduce the participation burden [15,17].

The e-questionnaire was launched online on 15 May 2021 and remained open to participants until 15 October 2021. Questionnaire distribution was performed as previously described [15,17]. Direct messaging and social media post dissemination strategies were adopted. Several social media channels, including WhatsApp, Facebook and Twitter, were differentially used to reach specific target audiences (Figure 1). Descriptive statistics were performed, taking advantage of the Survey Monkey and Microsoft Excel data analysis tools. Graphs were created with GraphPad Prism (version 8, San Diego, CA, USA).

### 2.2. Development of Plain Language CMGs Based on Participatory Health Literacy

This study was based on the first-ever published CMGs for CDG, the “International clinical guidelines for the management of phosphomannomutase 2-congenital disorders of glycosylation: Diagnosis, treatment and follow up” published in 2019 [20].

**Figure 2:**
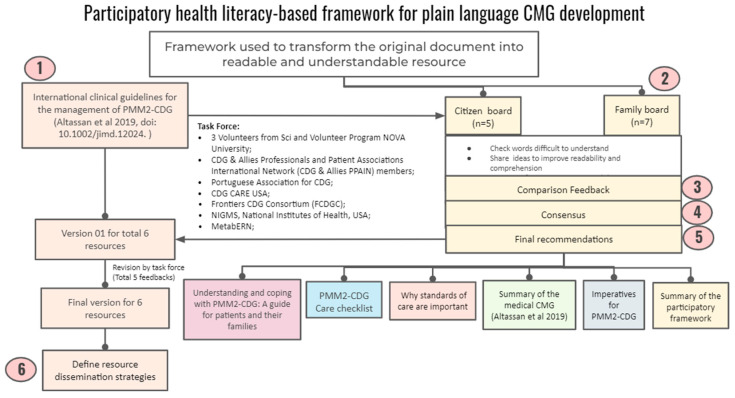
Framework for developing plain CMGs based on CDG community’s needs/preferences and health literacy principles.

Following up on the quantitative results obtained through the e-questionnaire, we created a participatory health literacy-based framework for developing plain language CMGs using the PMM2-CDG CMGs as a template. Figure 2 shows the different framework phases, stakeholders, and proposed outputs.

Briefly, a task force of two Advisory Boards was set up. The citizen board encompassed CDG expert professionals and biomedical bachelor’s degree university students (*n* = 5), while the other was composed of CDG family members (*n* = 7). The identification and recruitment of the participants took place through contact with several institutions, such as POs (CDG CARE and APCDG), international networks (Frontiers in CDG Consortium and MetabERN), and academic (NOVA University Lisbon, Lisbon, Portugal) and health care (e.g., Mayo Clinic, Rochester, MN, USA, KU Leuven, Belgium) institutions,, to ensure diverse representation in terms of background, institutional affiliation, age and country coverage. We shared the PMM2-CDG CMGs with the participants via email and asked them to assess the CMGs according to three main activities. Firstly, they were asked to identify challenging terms to understand (e.g., medical jargon and technical terms). Secondly, ideas should be shared to improve readability and comprehension. Finally, the advisory board participants were requested to suggest strategies for communicating the new plain-language CMGs.

Four researchers analysed the feedback. Consensus was sought, and when this was not possible, we used the views agreed upon by most participants. Based on these, final recommendations were issued. They encompassed the development of six CMG-related resources, including the production of a plain language version of the PMM2-CDG CMGs, as well as resource distribution through social media channels and email lists to all the participating institutions. The task force performed five rounds of revision and then refined the co-created resources (Figure 2).

## 3. Results

### 3.1. E-Questionnaire Participants’ Academic Qualifications and Distribution

**Figure 3:**
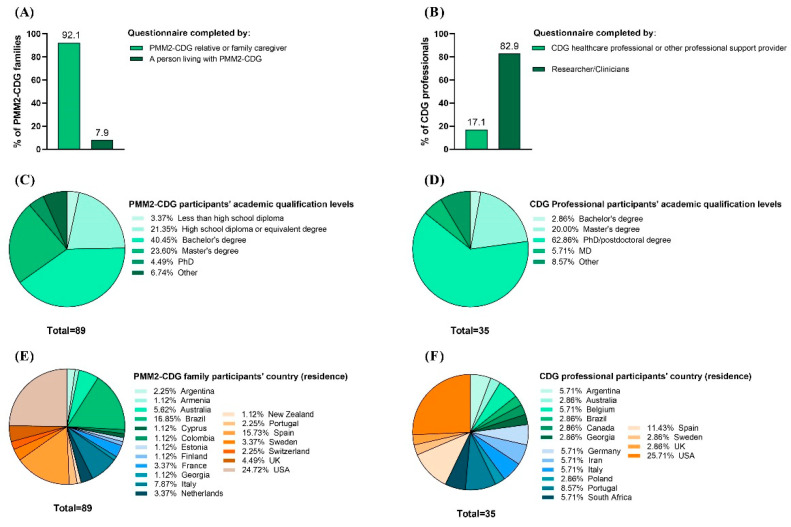
Characteristics of the CDG Journey Mapping e-Questionnaire respondents. It included PMM2-CDG family members and professional stakeholders. (**A**) Family and (**B**) professional respondents’ categories; (**C**) families’ and (**D**) professionals’ academic qualifications; (**E**) families’ and (**F**) professionals’ geographic distribution (per country of residence).

A total of 193 CDG patients and family members and 35 professionals completed the e-questionnaire. This study only included PMM2-CDG family stakeholders since they represented the largest CDG group with published CMGs, accounting for 46.1% (89 participants) of all of the e-questionnaire family/patient participants. Among the remaining 104 (53.9%) CDG family respondents, 85 reported a CDG other than PMM2-CDG and 19 were suspected to be duplicated responses and/or respondents without a final CDG diagnosis, being the reasons why they were excluded from this study. For the professionals, 100% of the participants were considered (Figure 3A,B). Most PMM2-CDG family respondents (92.1%) were relatives or family caregivers (Figure 3A). For the professionals, the majority (82.9%) were researchers and clinicians (Figure 3A). Both participant groups had high academic qualifications, with 68.5% of the PMM2-CDG family members holding at least a bachelor’s degree and 62.9% of the professionals holding a PhD or postdoctoral degree (Figure 3C,D). Additionally, participants were distributed worldwide, which underlines the international nature of the CDG family and professional communities (Figure 3E,F). CDG professional respondents displayed a varied institutional distribution, with the Mayo Clinic having the highest representation (Supplementary Figure S2).

### 3.2. The Perceptions of CMGs among CDG Families and Professionals

**Figure 4:**
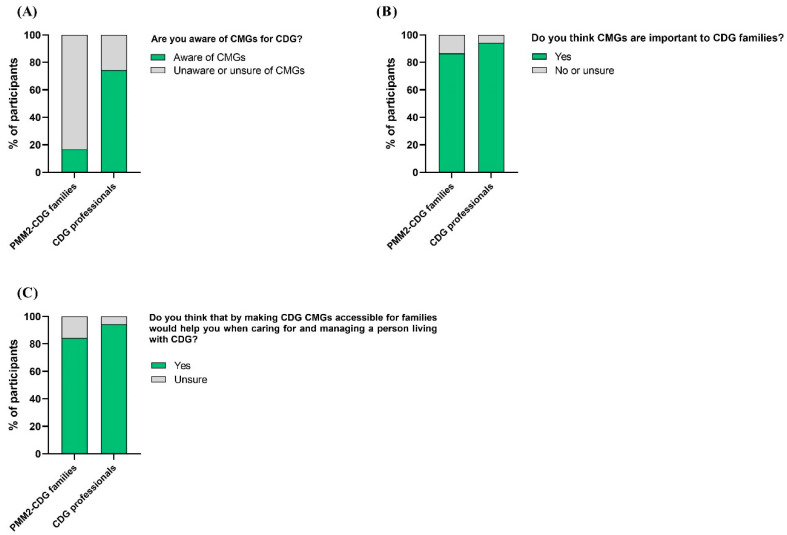
CMG recognition, perceived importance, and usefulness by CDG family and professional participants. (**A**) Families and professionals’ awareness of CMGs; (**B**) families and professionals attributed importance to CMGs; (**C**) CMG perceived usefulness by families and professionals in the care and management of CDG.

To fully understand the CDG community’s views on CMGs, participants of the CDG Journey Mapping e-Questionnaire were asked about their CMG-related perceptions, including recognition, perceived importance, and usefulness. Participants showed different levels of recognition of CMGs. PMM2-CDG family respondents were widely unaware or unsure of any CMGs (83.1%), versus only 25.7% of professionals (Figure 4A). However, families (86.5%) and professionals (94.3%) described CMGs as essential tools for families, explaining that making them accessible to families would help them in CDG care and management (Figure 4B,C).

### 3.3. Challenges and Solutions for the Comprehension and Accessibility of CMGs

**Figure 5:**
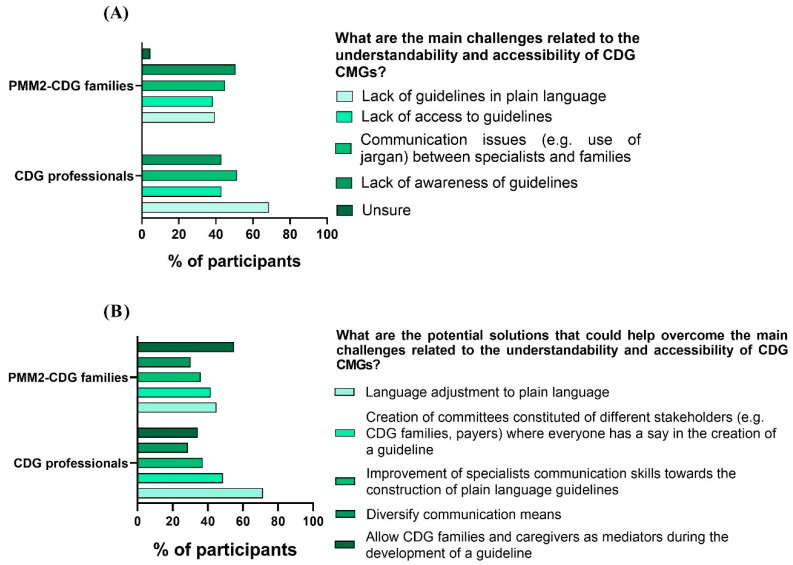
CMG challenges and solutions identified by CDG family and professional participants. (**A**) Families and professionals pinpointed CMG challenges; (**B**) families and professionals proposed CMG solutions.

**Figure 6:**
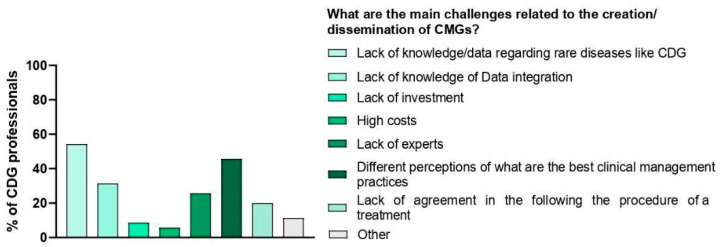
Main challenges related to the creation/dissemination of CMGs.

We also questioned e-questionnaire participants about the challenges they identified regarding the comprehensibility and accessibility of CMGs. The challenge that families pinpointed the most was the lack of awareness of CMGs (50.6%), followed by communication problems (e.g., the use of jargon) between families (44.9%) and professionals (51.4%). Professionals highlighted the lack of plain language guidelines (68.6%) as the most frequent obstacle to CMG understanding and access (Figure 5A). Interestingly, professionals also shared that the main obstacles they identified were the lack of data on CDG (54.3%) followed by the lack of agreement on the best clinical management practices (45.7%) (Figure 6).

For the solutions, families felt that the most impactful action would be to include family members in CMG development (55.1%), while professionals selected adapting CMGs to plain language (71.4%) as the most beneficial measure toward ensuring CMG comprehension and access (Figure 5B).

### 3.4. CMG-Related Resources Adapted to the CDG Community’s Needs and Preferences to Improve Comprehension and Accessibility

**Figure 7:**
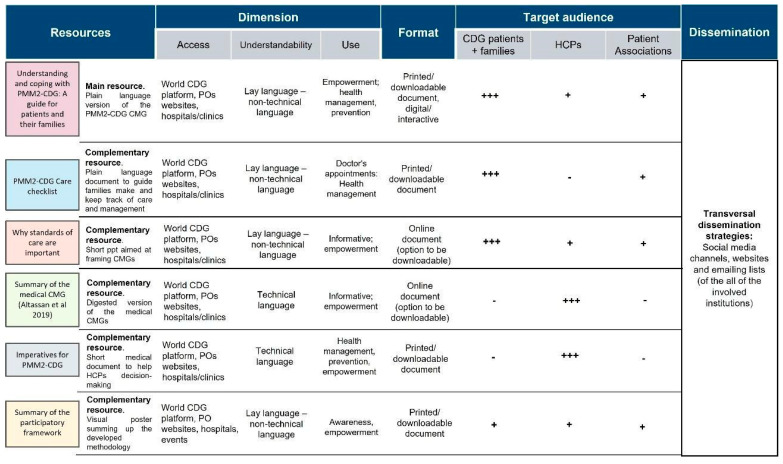
The six CMG-related resources co-created by the CDG family and professional communities. A tripartite category was adopted to identify the target audiences: +++: primary target audience; +: secondary/complementary target audience; -: untargeted audience; legend: HCP—health care professionals; POs—patient organisations.

Following the collection of quantitative data, which allowed us to determine the needs and experiences of the CDG community regarding CMGs, a protocol was designed and implemented to overcome identified challenges by adopting the proposed solutions. A participatory framework (involving CDG families and professionals) was created to improve CMG comprehensibility and accessibility, namely, through (1) the involvement of diverse stakeholders, including CDG family members in CMG development and distribution; (2) the creation of plain language versions for CDG CMG (beginning with PMM2-CDG CMGs); and (3) the design of a distribution strategy to ensure broader access to CMGs. The resulting co-created outcomes encompassed six CMG-related resources (see Figure 7).

Each resource was characterised in several dimensions (access, comprehension, and use), format (printed, digital) and target audience (CDG patients/families, HCP and POs).

The leading resource generated from this framework is “Understanding and coping with PMM2-CDG: A guide for patients and their families”, which is a plain language adaptation of the published PMM2-CDG CMGs [20]. The five additional resources aimed to complement this primary outcome by providing more summarised information and/or acting as clinical decision/care support tools for different target audiences. For example, the “Imperatives for PMM2-CDG” document is to guide clinician diagnostic and follow-up management strategies; whereas the “PMM2-CDG care checklist” is for families and POs and aims to assist them in tracking their medical appointments and tests/exams.

## 4. Discussion

This study tested and implemented an innovative, participatory methodology to answer families’ and professionals’ views on and experiences with CMGs. Multistakeholder, community-centric approaches create inclusive solutions that benefit all community members. These engagement and co-creation exercises present several advantages, including spurring innovation, improving decision quality, expanding credibility, increasing sustainability, and promoting community and capacity-building [26,27].

Indeed, participatory methodologies contributed to the development of educational materials for rare diseases [26], and the trust of the CDG community in POs is well documented [2,3,18,19]. Additionally, HCPs are the most reliable source of health information, and CDG families trust them when making infection- and vaccine-related decisions [17]. Trust in information sources is fundamental to adapt, change or endorse any health-related behaviour and/or action, including research participation [28,29,30]. Notably, POs and expert HCPs were involved in our project’s different steps and strategies, extending to framework implementation and resource development.

We began by collecting quantitative data from CDG families and professionals on their CMG perceptions, needs and preferences. Of note, PMM2-CDG families revealed poor CMG awareness levels, concordantly identifying lack of awareness of CMGs as the most frequent challenge followed by communication problems (e.g., jargon use) between themselves and professionals. PMM2-CDG family respondents strongly suggested involving family members during CMG development to overcome these problems. 

Plain language is effective and vital to increasing health literacy. Professionals highlighted the lack of guidelines in plain language as the most common obstacle, putting their creation forward as their top solution. Accordingly, communication competencies, including clear, assertive, positive and plain language, have been defined as one of the core missions of medical professionals [31,32,33].

We also collected a number of rules that would guide us on plain language writing in order to achieve a better understanding and an easy-to-visit document. Concerning the words that we should use: (1) identify who your readers are: who’s the main target of your work? (2) Write for the average reader using common, everyday words, but avoid slang. (3) Use a conversational tone instead of a more formal, bureaucratic one. (4) Avoid undefined technical terms. (5) Use personal pronouns such as “you”. (6) Use positive rather than negative words. (7) Avoid long strings of nouns. (8) Use “must” to express requirements instead of the ambiguous word “shall”. About the verbs that we should use: (1) use the active voice; (2) use action verbs; (3) use the simplest tense possible, simple present is best.

The structure and display of the content are also important features in creating simple language resources. The way we place the information is important for the understanding and the easy and quick consultation of these documents. Hence, the rules that guided us in this task were: (1) be direct, omit unnecessary details and avoid excess words. (2) Start your text with an introduction and a table of contents to help readers understand how it is organised. (3) Use headings to guide readers; the question-and-answer format is very helpful and you can organise information to answer these questions. (4) Use short paragraphs and sentences (average 15–20 words), each paragraph should have only one topic. Provide space between sections. (5) Use transitions to connect ideas, sentences and paragraphs. (6) Limit the number of fonts you use, one for headings and another for text. (7) Use images, tables and schemes to make complex information more understandable. (8) Use bullets, bold and italics to emphasise information.

Nevertheless, the development of plain language versions of medical and scientific documents is still in its early stages, especially CMGs [34]. The increasing importance attributed to patient/family caregiver empowerment, health care and research involvement, also evidenced by these study results, has boosted incentives and interest in designing and disseminating plain language resources [1,35,36]. Moreover, health literacy directly influences adequate health-promoting and preserving behaviours, the quality of health care, access to medical innovations and consequently patients’ quality of life and health care expenses and costs [11,33,35,37,38].

A co-creation framework was implemented, guided by an unmet need and opportunity analysis of CMG development and dissemination. Six complementary and population-targeted resources and a CDG community-tailored dissemination plan were proposed. The dissemination strategy used by the CDG community relied on digital channels, including social media and email lists from the participating institutions. These measures reinforce the CDG community’s preference for digital solutions, which may stem from the worldwide distribution of CDG families and professionals [15,17,18,19].

### 4.1. Study Strengths and Limitations

The high participation among CDG families, particularly PMM2-CDG family caregivers and patients, in this study aligned with the results of previous e-questionnaires and the evidence of a participatory and pro-research attitude in the CDG community [2,3,15,17,18]. However, professionals’ low level of participation is of note. This could be due to the lack of experience and/or tradition in recruiting CDG professionals in e-questionnaire-based studies. Consequently, these results point to the need to improve recruitment and engagement strategies with this CDG community group. Additionally, unlike the multilingual versions created for families, their e-questionnaire was only available in English, which might have negatively impacted participation. Nevertheless, both CDG families and professionals showed a worldwide distribution, similar to what was observed in previous studies [15,17,19], even though country diversity was higher among PMM2-CDG family stakeholders.

Additionally, both participant groups had high academic qualifications. On the one hand, this participation bias can positively influence data quality. On the other hand, it might not represent the needs of the broader CDG community. Despite higher education being related to higher literacy levels, health literacy is also influenced by age, financial status, social connectedness, disability and contextual settings [37,38,39]. This reflects the multidimensional and ever-evolving nature of health literacy [10,40,41].

Notably, developing CMG in plain language is also an innovative project entailing numerous resources, including teamwork and funding.

### 4.2. Future Perspectives

The flexibility and adjustability of the developed framework open the door to its adaptation to other CDG CMGs. Phosphoglucomutase 1 (PGM1)-CDG and mannose 6-phosphate isomerase (MPI)-CDG already have published CMGs [21,22]; hence, the creation of accessible and understandable plain language resources using the methodology described in the present study is already underway. CMG development for rare diseases faces many challenges. In particular, CDG data scarcity and the lack of agreement on the best clinical management practice are barriers also reported for other rare diseases [2,4,7,42,43]. Hence, these shared challenges reinforce the suitability of extending this methodology to other rare disease communities.

A dedicated, free section is available on the World CDG Organisation platform (https://worldcdg.org (accessed on 15 July 2021)) to ensure broader accessibility to the plain language CMGs and all additional resources. The multilanguage translation of CMG-related resources is a future priority since the CDG community is eminently international and multilingual [15,17,18,19].

Finally, diversifying the format of modern resources, such as podcasts, and designing good distribution campaigns by further implementing the framework and involving all stakeholders will take place.

## 5. Conclusions

CMGs are essential clinical decision support tools for both HCPs and families. We established a reproducible health literacy-based environment to co-create clear and understandable CMGs through a participatory methodology responding to the different views of stakeholders. Although this work has been piloted in the PMM2-CDG community, it can benefit all CDG community members and ultimately improve the quality of life of CDG patients. Moreover, it holds tremendous potential for other CMGs benefiting other rare disease communities.

## Supplementary Materials

The following supporting information can be downloaded at: https://www.mdpi.com/article/10.3390/ijerph192013506/s1, Figure S1: CDG Journey Mapping structure. The sections specific for CDG families are coloured in light green while the professional-specific sections are represented in dark green. Sections common to both groups are shown in blue. The CMG section is highlighted in red.; Figure S2: Affiliations of the professionals who completed the e-questionnaire.
