# Supplementary material for: A Participatory Framework for Plain Language Clinical Management Guideline Development
Source: Int J Environ Res Public Health. 2022 Oct 19;19(20):13506. doi: 10.3390/ijerph192013506 (PMC9603256; doi:10.3390/ijerph192013506)
Supplement: Supplementary file 1 [file ijerph-19-13506-s001.zip › ijerph-1865738-supplementary.pdf]

Supplementary material

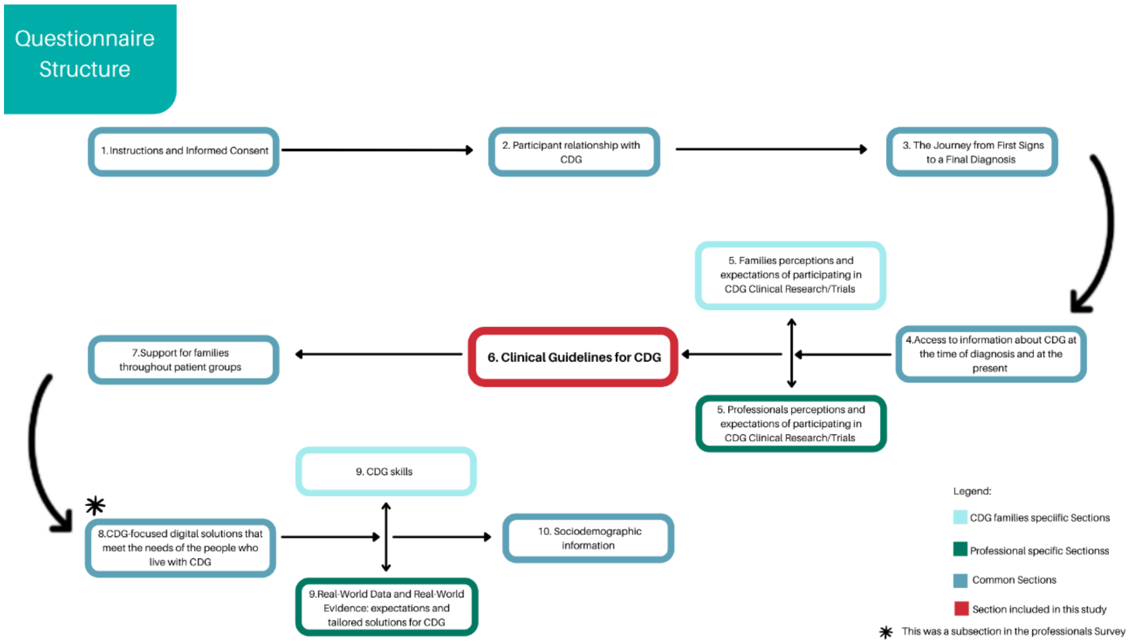

Supplementary Figure S1. **CDG Journey Mapping structure.** The sections specific for CDG families are coloured in light green while the professional-specific sections are represented in dark green. Sections common to both groups are shown in blue. The CMG section is highlighted in red.

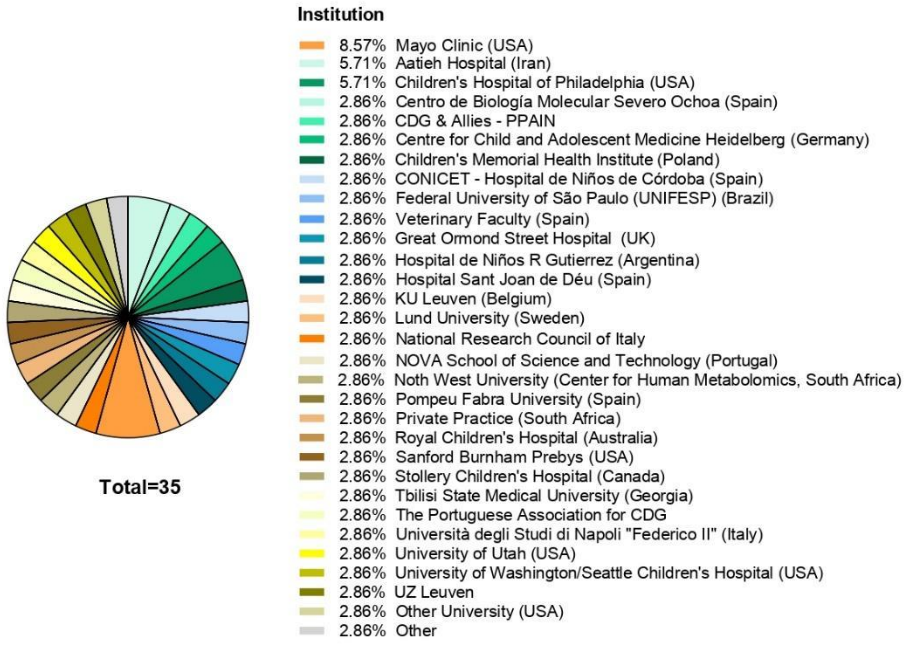

Supplementary Figure S2. Affiliations of the professionals who completed the e-questionnaire.
